# Abnormal functional connectivity of the striatum in first‐episode drug‐naive early‐onset Schizophrenia

**DOI:** 10.1002/brb3.2535

**Published:** 2022-04-05

**Authors:** Yan Zhang, Yue Peng, Yichen Song, Youqi Zhou, Sen Zhang, Ge Yang, Yongfeng Yang, Wenqiang Li, Weihua Yue, Luxian Lv, Dai Zhang

**Affiliations:** ^1^ Psychiatry Institute of Mental Health/Peking University Sixth Hospital Peking University Beijing China; ^2^ Henan Mental Hospital The Second Affiliated Hospital of Xinxiang Medical University Xinxiang China; ^3^ Henan Key Lab of Biological Psychiatry, Xinxiang Medical University Xinxiang China; ^4^ International Joint Research Laboratory for Psychiatry and Neuroscience of Henan Xinxiang China; ^5^ Department of Pediatric Rehabilitation Medicine The Fifth Affiliated Hospital of Zhengzhou University Zhengzhou Henan China; ^6^ Child and Adolescent Psychiatry Department Mental Health Center of Shantou University Shantou Guangdong China

**Keywords:** early‐onset schizophrenia, functional connectivity, region of interest, resting state, striatum

## Abstract

Abnormal brain network connectivity is strongly implicated in the pathogenesis of schizophrenia. The striatum, consisting of the caudate and putamen, is the major treatment target for antipsychotics, the primary treatments for schizophrenia; however, there are few studies on the functional connectivity (FC) of striatum in drug‐naive early‐onset schizophrenia (EOS) patients. We examined the FC values of the caudate nucleus and putamen with whole brain by resting‐state functional magnetic resonance imaging (RS‐fMRI) and the associations with indices of clinical severity. Patients demonstrated abnormal FC between subregions of the putamen and both the visual network (left middle occipital gyrus) and default mode network (bilateral anterior cingulate, left superior frontal, and right middle frontal gyri). Furthermore, FC between dorsorostral putamen and left superior frontal gyrus correlated with both positive symptom subscore and total score on the Positive and Negative Syndrome Scale (PANSS). These findings demonstrate abnormal FC between the striatum and other brain areas even in the early stages of schizophrenia, supporting neurodevelopmental disruption in disease etiology and expression.

## Graphical abstract

We examined the functional connectivity of the caudate nucleus and putamen with whole brain in early‐onset schizophrenia by resting‐state functional magnetic resonance imaging and the associations with indices of clinical severity.

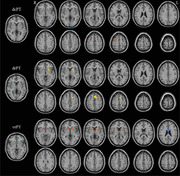

## 1 INTRODUCTION

Schizophrenia is a debilitating mental disorder with worldwide prevalence of about 1%. A plethora of drugs are available targeting specific symptoms, but disease course is characterized by high recurrence rate and poor compliance due to intolerable side effects (Insel, 2010). Furthermore, etiology is uncertain so there are no therapies targeting the underlying pathogenic mechanisms. Symptoms usually emerge in adolescence and early adulthood (van Os & Kapur, 2009), suggesting neurodevelopmental abnormalities. Common symptoms include hallucinations, delusions, socially inappropriate behaviors, apathy, social withdrawal, and cognitive impairment (Marin, 2012; Picchioni & Murray, 2007). It is widely accepted that early diagnosis and treatment improve prognosis (Abidi, 2013), underscoring the importance of identifying biomarkers in the prodromal period prior to symptom onset.

The caudate nucleus and putamen, basal ganglia structures collectively termed the striatum, are involved in motor control and reward‐dependent behavior among other functions (Parent, 1990). (See Supporting Information). The striatum is also a major target for schizophrenia treatments, including dopamine receptor antagonists (Fornito et al., 2013; Li, Zalesky et al., 2020; McCutcheon et al., 2019). Previous positron emission tomography (PET) studies have shown that the largest dopamine abnormalities occur in the dorsal striatum (Weinstein et al., 2017), and that dopamine synthesis in the striatum is significantly higher among people at greater risk of mental illness (Howes et al., 2011; Mizrahi et al., 2014). The “dopamine hypothesis” (Guillin et al., 2007; Howes & Kapur, 2009) suggests that schizophrenia is caused by hyperdopaminergic function in the striatum and hypodopaminergic function in the prefrontal lobe. Antipsychotics are the most effective treatment for schizophrenia, and most antagonize dopamine receptors in the striatum (Leucht et al., 2013; Meltzer, 2017). The functional connectivity (FC) of the striatum is substantially influenced by dopaminergic transmission (Seeman & Lee, 1975). Previous studies have found abnormal FC between the striatum and cortex in patients with mental illness (Fornito et al., 2013) . This abnormal FC can be used as a potential biomarker for schizophrenia and may even predict the therapeutic efficacy of antipsychotics (Sarpal et al., 2015) . Alterations in striatal functional networks have been found not only in schizophrenia patients (Martino et al., 2018; McCutcheon et al., 2019), but also in first‐degree relatives (Fornito et al., 2013; Li, Yan et al., 2018). Moreover, abnormal corticostriatal FC is associated with clinical symptom severity (Tu et al., 2012), while symptom improvement by antipsychotics is associated with amelioration of these FC abnormalities (Sarpal et al., 2015).

Morphological changes of striatum have been investigated as potential biomarkers for schizophrenia risk and prognosis (Zampieri et al., 2014) . In addition, functional changes revealed by neuroimaging may provide useful markers for diagnosis or prognosis. Resting‐state functional magnetic resonance imaging (RS‐fMRI) is a noninvasive imaging modality that can indirectly reveal task‐independent neural activity patterns within regions (Logothetis & Wandell, 2004) and the temporal correlation of activity among regions (i.e., within functional brain networks) (Logothetis, 2008). Resting‐state fMRI studies hold great potential for elucidating the pathophysiological mechanisms of schizophrenia (Liao et al., 2012; Rotarska‐Jagiela et al., 2010; Zhou et al., 2007). Indeed, such studies have demonstrated abnormal FC values in schizophrenia (Pettersson‐Yeo et al., 2011; Wheeler et al., 2015), some of which are directly correlated with clinical symptom severity (Sarpal et al., 2015). However, there are inconsistencies among such studies, with some reporting weaker FC (Liang et al., 2006; Pettersson‐Yeo et al., 2011), others stronger FC (Liu et al., 2012), and still others both stronger and weaker FC across different pathways (Damaraju et al., 2014; Skudlarski et al., 2010; Woodward et al., 2011; Zhuo et al., 2018). These discrepancies may be related to the use of antipsychotics, disease duration, analytics and statistical methods, and sample size among other factors. Therefore, this study focused on first‐episode drug‐naive early‐onset schizophrenia (EOS) to reduce the influence of confounding factors.

Early‐onset schizophrenia (EOS) is defined by onset age younger than 18 years, while very early‐onset schizophrenia, which is relatively rare, is defined by onset before 13 years of age (Abidi, 2013). The etiology and pathogenesis of schizophrenia remain controversial, but it is widely accepted that multiple mechanisms contribute, including neurodevelopmental defects. This theory holds that neurogenesis, synaptogenesis, pruning, and circuit formation during adolescence and early adulthood may be disrupted due to genetic factors, environmental factors, or a gene × environment interaction, leading to the progressive emergence of symptoms (Fatemi & Folsom, 2009). Compared to adult‐onset schizophrenia, EOS is less influenced by environmental factors in adulthood, and neurodevelopmental abnormalities appear to be predominant (Murray et al., 1992). Therefore, studies of EOS may reveal the underlying neurodevelopmental mechanisms of schizophrenia.

In this study, we compared striatal FC between first‐episode drug‐naive early‐onset schizophrenics and health controls using the caudate nucleus and putamen as regions of interest. Given the contributions of striatal hyperdopaminergic signaling and FC abnormalities to psychotic symptoms, we hypothesized that EOS patients would exhibit abnormal FC values between striatum and other brain regions at rest and that these abnormal FC values would be associated with positive symptoms. Our aim is to investigate the characteristics and clinical significance of striatal FC in EOS.

## 2 MATERIALS AND METHOD

### 2.1 Study subjects

The study involved 183 right‐handed subjects, 76 healthy controls recruited from the local community by advertising, and 107 first‐episode drug‐naïve EOS patients recruited from the Second Affiliated Hospital of Xinxiang Medical University. Inclusion criteria were (1) 13 to 18 years old, (2) onset age between 13 and 18 years, (3) disease duration less than 2 years, (4) disease confirmed by two psychiatrists according to Diagnostic and Statistical Manual of Mental Disorders, Fourth Edition–Text Revision (DSM‐IV‐TR) criteria, (5) no current or prior antipsychotic medication use, (6) no comorbid Axis I diagnosis, (7) intelligence quotient (IQ) greater than 70, and (8) more than 6 years of education. All patients were assessed for symptom severity using the Positive and Negative Syndrome Scale (PANSS). The healthy control group was matched for age, sex ratio, and years of education. None of these control patients had direct relatives in the past three generations with a history of mental illness. The exclusion criteria for all participants were as follows: traumatic brain injury leading to loss of consciousness, neuromuscular disorders, neurological disorders, alcohol or drug abuse, mental retardation, and contraindications for MRI scanning.

The study protocol was approved by the Ethics Committee of the Second Affiliated Hospital of Xinxiang Medical University. Written informed consent was obtained from each participant or from the participant's parents or legal guardians.

### 2.2 Image acquisition

Imaging data were obtained using the 3.0 Tesla Siemens TIM TRIO system (Siemens, Erlangen, Germany). T1‐weighted images were obtained in an axial orientation using the following parameters: repetition time/echo time (TR/TE) = 2530/2.43 ms, matrix = 256 × 256, voxel size = 1 × 1 × 1 mm^3^, and flip angle = 7°. There were 158 slices without interlaminar space. Functional images were collected using an echo plane sequence with the following parameters: TR/TE = 2000/30 ms, slice = 33, matrix = 64 × 64, flip angle = 90°, field of view = 220 × 220 mm^2^, interlaminar clearance = 0.6 mm, and voxel size = 3.44 × 3.44 × 4 mm^3^. The fMRI scan lasted for 6 min and 240 volumes were obtained.

### 2.3 The fMRI preprocessing

Statistical Parameter Mapping 12 (http://www.fil.ion.ucl.ac.uk/spm) and DPARSFA software were used to preprocess fMRI data (Chao‐Gan & Yu‐Feng, 2010) . The first 10 volumes of each functional time series were discarded to mitigate influences of scanning noise adaptation. The remaining fMRI images were corrected for interslice acquisition delays and head movements. Briefly, mean framewise translational and rotational displacement (FD) values due to head motion were calculated according to a formula described previously (Jenkinson et al., 2002; Power et al., 2012). Images were processed using the Diffeomorphic Anatomical Registration using Exponential Lie Algebra (DARTEL) tool, and the resulting images normalized to Montreal Neurological Institute (MNI) space with 3 × 3 × 3 mm^3^ voxels (Klein et al., 2009). Then, regression analysis was performed on the Friston‐24 motion parameters, cerebrospinal fluid, white matter signals, and other linear interference signals. The image space was smoothed using a 6‐mm full‐width at half‐maximum isotropic Gaussian kernel and 0.01‒0.1 Hz bandpass filtering.

### 2.4 FC analysis

As described in previous studies (Di Martino et al., 2008; Fornito et al., 2013; Postuma & Dagher, 2006), three areas of the bilateral putamen and three areas of the bilateral caudate along the dorsal‐ventral axis, each 3.5 ‐mm in radius, were defined as seed regions for FC analysis. For the putamen, a plane at *z* = 2 mm distinguished the dorsocaudal putamen (dcPT) (*x* = 28, *y* = 1, *z* = 3) and dorsorostral putamen (drPT) (*x* = 25, *y* = 8, *z* = 6) from the ventrorostral putamen (vrPT) (*x* = 20, *y* = 12, *z* = 3). For the caudate, a horizontal plane at *z* = 7 mm distinguished the dorsal caudate (DC) (*x* = 13, *y* = 15, *z* = 9) from the superior ventral caudate (sVC) (*x* = 10, *y* = 15, *z* = 0) and inferior ventral caudate (iVC) (*x* = 9, *y* = 9, *z* = 8). Pearson's correlation coefficients (*r* values) were calculated between these six seed regions and whole brain voxels and then converted to *Z* values using Fisher's transform. For simplicity, we focused on average results from the left and right seed regions.

### 2.5 Statistical analysis

Demographic data were compared between groups by chi‐square test and two‐sample *t*‐test as indicated. Functional connectivity maps were generated for both groups using Statistical Parameter Mapping 12 software to identify abnormalities in EOS patients. Two‐sample *t*‐test was used to compare EOS patients and healthy controls with gray masks. Mean FDs were used as confounding factors in the calculation. The Gaussian random field (GRF) approach was utilized to adjust *p* values at the cluster level, with *p* < .05 defined as cluster‐level significance and *p* < .001 as voxel‐level significance. Pearson's correlation coefficients were also calculated between each individual patient PANSS score and *Z* values to estimate the influence of FC on clinical symptom severity.

## 3 RESULTS

### 3.1 Demographic and clinical characteristics

Of 112 patients with EOS and 78 healthy controls, data from five patients and two controls were excluded due to excessive head movement during the scan (mean FD < 0.2), so data from 107 patients and 76 controls were included in the final analyses. There were no significant differences in age, sex ratio, framewise displacement, and educational level between groups (all *p* > .05). The demographic and clinical characteristics of the study population are summarized in Table 1.

**TABLE 1:** Demographic and clinical characteristics of participants

	EOS (*n* = 107)	HC (*n* = 76)	*t*/*χ* ^2^	*p*
age (years)	15.33 ± 1.62	15.43 ± 1.86	−0.415	.679
education (years)	9.17 ± 1.86	9.01 ± 2.00	0.540	.590
sex	45 m/62 f	30 m/46 f	0.123	.726
FD (mm)	0.066 ± 0.059	0.061 ± 0.031	0.621	.535
duration (months)	5.47 ± 6.82	−	−	−
PANSS				
Positive	21.56 ± 5.49			
Negative	20.03 ± 7.45			
General	37.22 ± 6.95			
Total	81.76 ± 13.44			

EOS, early‐onset schizophrenia; HC, healthy controls; FD: framewise displacement; PANSS, Positive and Negative Syndrome Scale.

### 3.2 FC between two groups

**FIGURE 1:**
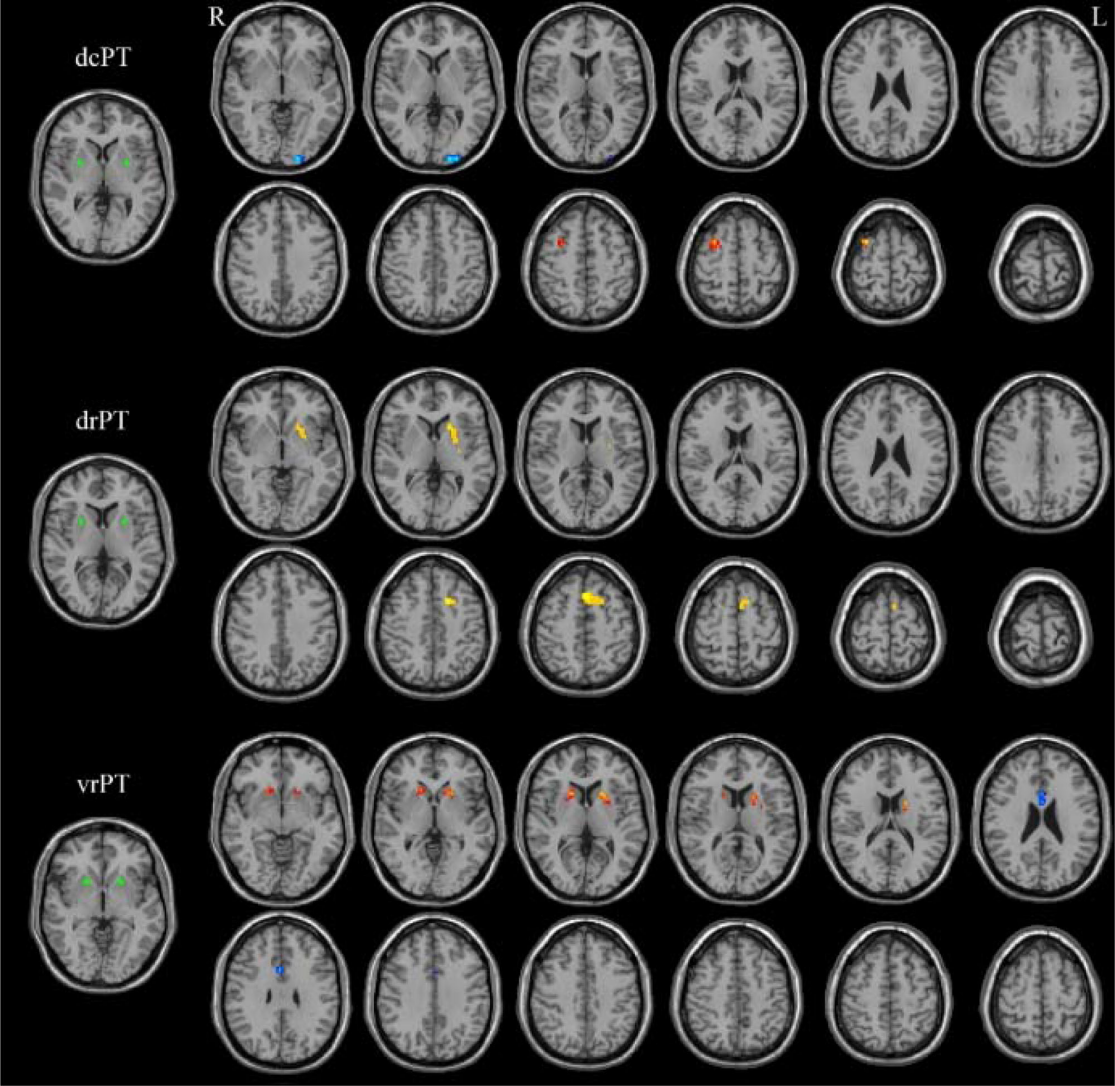
Differences in functional connectivity (FC) of the dorsocaudal putamen (dcPT), dorsorostral putamen (drPT), and ventrorostral putamen (vrPT) between early‐onset schizophrenia (EOS) patients and healthy controls. Warm and cool colors denote stronger and weaker FC, respectively, in EOS patients

Patients with EOS presented with stronger FC between the dorsorostral putamen (drPT) and left superior frontal gyrus, weaker FC between the ventrorostral putamen (vrPT) and bilateral anterior cingulate gyrus (ACC), weaker FC between the dorsocaudal putamen (dcPT) and left middle occipital gyrus, and stronger FC between the dcPT and the right middle frontal gyrus (all voxel level *p* < .001, cluster level *p* < .05, GRF corrected) (see Table 2 and Figure 1).

**TABLE 2:** Differences in functional connectivity between EOS patients and healthy controls

		Peak (MNI)			
Anatomical regions	Voxel	*X*	*Y*	*Z*	*t* Value
**Decreased in patients with EOS**					
L middle occipital gyrus^a^	64	−18	−99	0	−4.42
Bilateral anterior Cingulate^c^	45	3	12	27	−4.29
**Increased in patients with EOS**					
R middle frontal gyrus^a^	58	30	6	63	4.53
L superior frontal gyrus^b^	145	−3	18	54	4.99
L **putamen** ^b^	91	−21	15	0	4.73
R **caudate** ^c^	47	18	18	6	4.83
L **putamen** ^c^	73	−18	15	9	5.17

^a^ Dorsocaudal putamen (dcPT) as the seed region.

^b^ Ventrorostral putamen (vrPT) as the seed region; voxel‐level *p* < .001; cluster significance: *p* < .05, GRF corrected.

^c^ Dorsorostral putamen (drPT) as the seed region.

MNI, Montreal Neurological Institute; L, left; R, right.

### 3.3 Relationship between brain function and psychopathology

**FIGURE 2:**
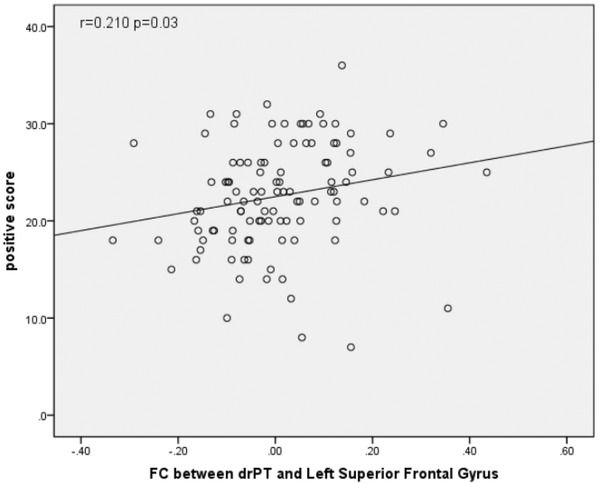
Correlation of PANSS positive symptom subscore with functional connectivity (FC) between the dorsorostral putamen (drPT) and superior frontal gyrus

**FIGURE 3:**
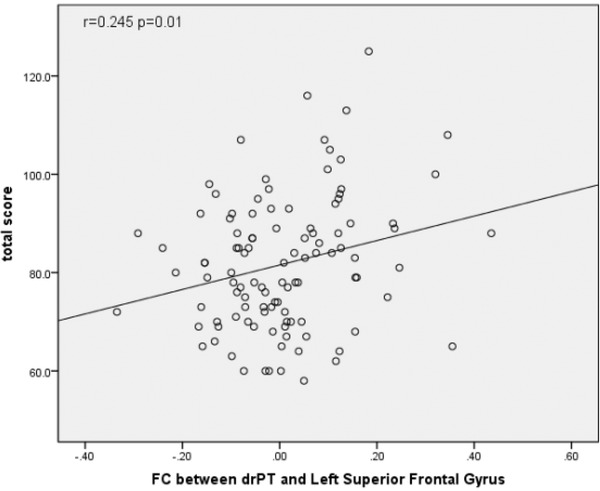
Correlation of PANSS total score with functional connectivity (FC) between the dorsorostral putamen (drPT) and superior frontal gyrus

The FC between drPT and left superior frontal gyrus was correlated with PANSS positive score (*r* = .210, *p* = .03) (Figure 2) and total score (*r* = .245, *p* = .01) (Figure 3). No other significant correlations were detected between FC values and PANSS scores.

## 4 DISCUSSION

To our knowledge, this is the first study to demonstrate abnormal striatal FC in treatment‐naïve early‐onset schizophrenia. Patients exhibited both stronger FC values (drPT to left superior frontal gyrus and dcPT to right middle frontal gyrus) and weaker FC values (vrPT to bilateral ACC and dcPT to left middle occipital gyrus). The ACC, superior frontal gyrus, and middle frontal gyrus are components of the default mode network (DMN) underlying internal mental states (Buckner & DiNicola, 2019; Zhao et al., 2018), while the middle occipital gyrus is a key component of the visual network (VN) (Li, Lu et al., 2020; Zhao et al., 2018). Concomitant disruption of these networks in the earliest stages of schizophrenia may contribute to psychotic symptoms.

The middle occipital gyrus is involved in visual and emotional processing (Lang et al., 1998), social cognition (Pelphrey et al., 2003), and working memory (Stablein et al., 2016). Previous studies have found structural abnormalities in the occipital lobe of schizophrenia patients (Guo et al., 2013; Onitsuka et al., 2007) and associations between these abnormalities and visual hallucinations (Fujimoto et al., 2013). In addition, abnormalities in VN core brain regions have been detected in both schizophrenia patients (Zhou et al., 2019) and high risk groups (Li, Lyu et al., 2018). Two previous studies also reported weaker connectivity between the VN and striatum in adult patients compared to matched controls (Koch et al., 2014; Yu et al., 2017), in accord with the current findings. The striatum is a major target of the dopaminergic reward system (Koch et al., 2014), which in addition to influences on reward‐dependent behavior can regulate visual processing (Hickey et al., 2010). The decrease in FC between dcPT and left middle occipital gyrus may be related in perceptual disturbances and poor judgment. The VN is also strongly related to emotion processing (Lang et al., 1998) and cognition (Pelphrey et al., 2003), and distorted visual perception may contribute to the negative affect and cognitive deficits of schizophrenia (Sterzer et al., 2011).

The DMN is responsible for monitoring the external environment at rest (Raichle & Snyder, 2007) and for generating internal mental states (Hahn et al., 2007). Numerous studies have found abnormal FC within the DMN of both schizophrenia patients (Guo et al., 2015; Peng et al., 2021; Zhang et al., 2020) and their first‐degree relatives (Ma et al., 2019). Moreover, antipsychotics can normalize aberrant DMN FC in schizophrenia (Guo et al., 2017), suggesting that DMN dysfunction is an early core pathogenic mechanism. Dopamine release is also abnormal in the striatum of schizophrenia patients (Howes et al., 2009; Kegeles et al., 2010), and dopamine transmission modulates DMN activity (Braskie et al., 2011; Tomasi et al., 2009). Several studies have suggested the presence of a striatal‒DMN loop in the human brain (Braskie et al., 2011) that is disrupted in schizophrenia (Salvador et al., 2010; Wang et al., 2015) and associated with clinical symptoms (Wang et al., 2015). This abnormal functional association between the DMN and striatum has also been found among individual at high risk of schizophrenia (Hua et al., 2019) and other diseases such as mild behavioral impairment (Lang et al., 2020), depression (Hua et al., 2019; Paul et al., 2019), and primary insomnia (Wang et al., 2018). The current study identified three distinct pathways showing aberrant functional connectivity with the DMN. In according with our findings, Huang et al. (2010) found significantly reduced low frequency fluctuation (ALFF) amplitude in medial prefrontal lobe and increased ALFF in the putamen of adults with first‐episode untreated schizophrenia, and suggested that these ALFF abnormalities may occur in the early stage of schizophrenia. Our study demonstrated possible weakening of this striatum‐DMN loop in the early stages of schizophrenia. The striatum integrates information associated with reward (Haber, 2016), while the DMN is involved in sensory information processing (Hahn et al., 2007), so abnormal FC may disrupt the normal influences of sensory stimuli on reward‐dependent behavior by impairing the realistic evaluation and comprehension of external events. Previous studies have found that there were volume changes in ACC (Vercammen et al., 2010), putamen, the superior and middle frontal gyrus (Yasuda et al., 2020) in schizophrenic patients, and the structural changes may affect their function. In addition, Chen and his colleagues (Chen et al., 2020) found increased ReHo and fALFF in putamen and abnormal FC between putamen and ACC, superior and middle frontal gyri in schizophrenia, which supported our results. The putamen belonged to striato‐pallido‐thalamo‐cortical circuits, with motivational and emotional processing and cognitive and executive functions (Haber, 2003).The ACC is related to higher cognition, emotion, pain and desire, etc. (Weston, 2012). Weakened FC between vrPT and ACC may lead to impairment of cognitive function, emotional abnormalities, and loss of motivation, which are common manifestations of schizophrenia. Both the middle frontal gyrus (Menon, 2011) and superior frontal gyrus (Wolf et al., 2008) are related to cognition. Our study found that increased FC occurred in dcPT and the right middle frontal gyrus, drPT and left superior frontal gyrus. The hyperconnectivity between these brain regions resulted in excessive prominence signals (Whitfield‐Gabrieli et al., 2009). This may be one of the reasons for the disorder of thinking in schizophrenics.

The FC between drPT and left superior frontal gyrus of the prefrontal lobe was positively correlated with PANSS positive score and total score. The connection between striatum and prefrontal lobe exhibits functional (Zhou et al., 2015) and structural (Bracht et al., 2014; Levitt et al., 2017; Levitt et al., 2020) abnormalities in schizophrenia patients. Abnormal connections in the prefrontal cortex (Bluhm et al., 2007; Garrity et al., 2007) and functional activity of the striatum (Sorg et al., 2013) are associated with the positive symptoms of schizophrenia. Moreover, abnormal frontostriatal function has also been found in high‐risk groups (Fornito et al., 2013), again consistent with our results. In contrast however, abnormalities in the frontostriatal pathway have also been implicated in negative symptoms (Kaladjian et al., 2015; Shukla et al., 2019), possibly reflecting heterogeneity of sample populations or different analytic methods, including different seed regions. Nonetheless, these findings suggest that abnormally stronger and weaker connectivity within frontostriatal pathways may contribute to the clinical symptoms of EOS.

## 5 LIMITATIONS

Our study had several limitations. First, schizophrenia symptoms are highly heterogeneous, reflecting distinct underlying pathomechanisms (Koutsouleris et al., 2008; Vanes et al., 2019). However, this study did not examine patient subgroups, which may have obscured other FC abnormalities or associations with clinical characteristics. Second, we focused only on FC abnormalities between the striatum and other brain regions. Third, there was no follow‐up to examine further changes associated with disease progression or drug treatment response. In our subsequent studies, the homogeneity of the sample should be optimized, participants should be followed up, and additional analytical methods used to further explore the contributions of aberrant FC to EOS.

Despite these limitations, we found distinct FC abnormalities between the striatum and both the DMN and VN in early stage, drug‐naive EOS patients, and one of these abnormalities was associated with clinical symptoms. These FC changes may reflect altered regulation of functional networks by dopamine signaling. Collectively, our research highlighted the importance of striatal interactions with other brain networks, including the DMN and VN, in the pathogenesis of schizophrenia (See Supporting Information).

## FUNDING

National Natural Science Foundation of China (to LX‐L: 81971252; to WQ‐L: U1904130), Major science and technology projects of Henan Province (to WH‐Y: 201300310200), Science and technology research project of Henan Province (to YF‐Y: 212102310589), Natural Science Foundation of Henan Province (to Y‐Z: 202300410318), Medical Science and Technology Research Project of Henan Province (to Y‐Z: HGJ20190479), Open Project of Henan Key Lab of Biological Psychiatry (to Y‐Z: ZDSYS2020003).

## CONFLICT OF INTEREST

None of the authors has any actual or potential conflict of interest to this study.

## Supporting information



SUPPORTING INFORMATION

## Data Availability

The date that support the findings of this study are available from the corresponding author upon reasonable request.
