# Supplementary figures and images for: Abnormal functional connectivity of the striatum in first‐episode drug‐naive early‐onset Schizophrenia
Source: Brain Behav. 2022 Apr 5;12(5):e2535. doi: 10.1002/brb3.2535 (PMC9120884; doi:10.1002/brb3.2535)

**Supplementary material**


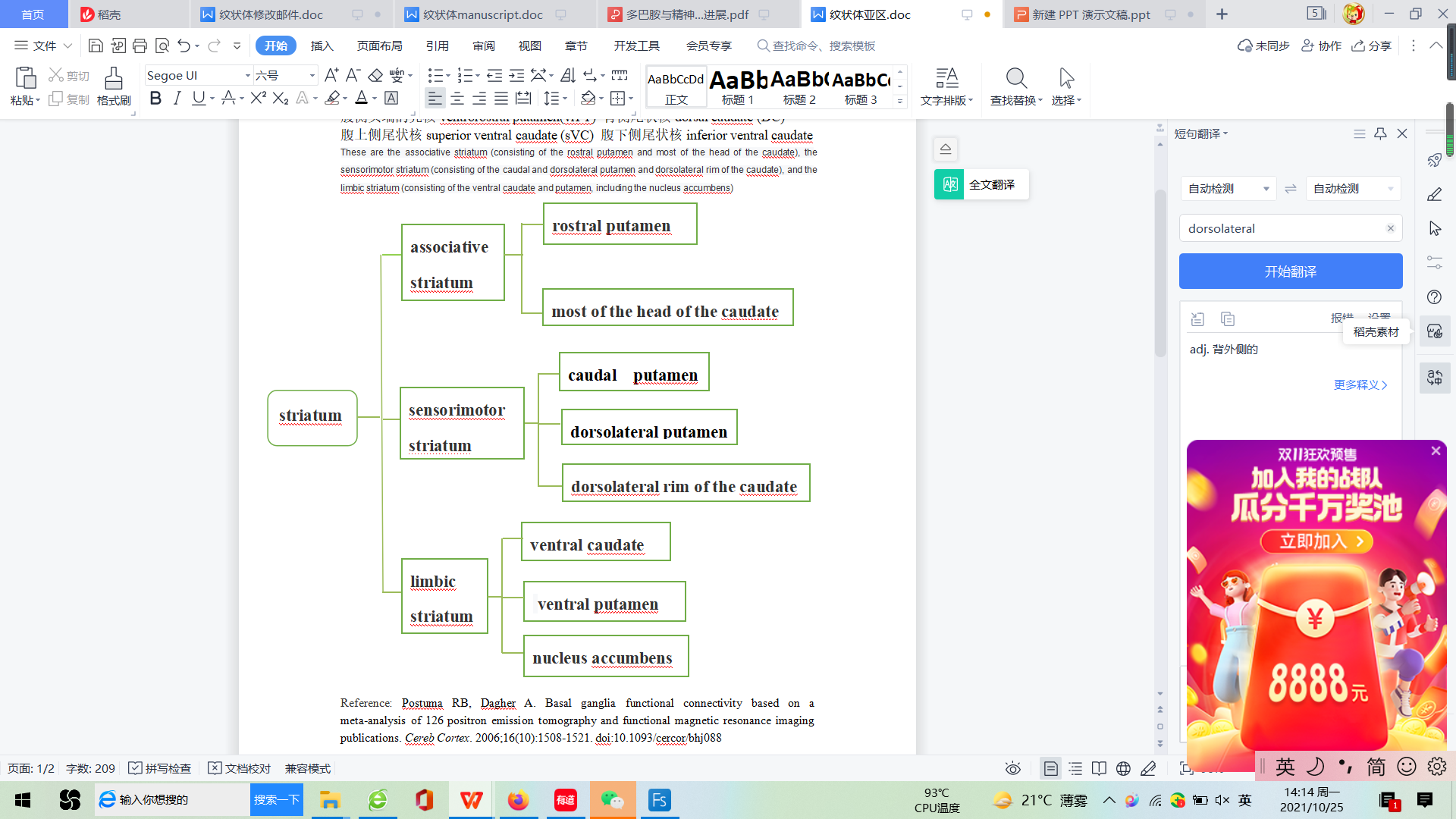

Supplement: Supplementary file 1 — SUPPORTING INFORMATION [file BRB3-12-e2535-s001.docx]
